# Dose-Dependent Efficacy of Ulinastatin in Improving 28-Day and 30-Day Survival Rates in Sepsis: A Systematic Review and Meta-Analysis

**DOI:** 10.3390/jcm15072682

**Published:** 2026-04-02

**Authors:** Qian Xiao, Rong Yang, Chuanying Huang

**Affiliations:** 1 Department of Emergency Medicine, West China Hospital of Sichuan University, Chengdu 610041, China; xiaoqian@wchscu.cn; 2 General Practice Ward/International Medical Center Ward, General Practice Medical Center, West China Hospital of Sichuan University, Chengdu 610041, China; yr1567@wchscu.cn

**Keywords:** sepsis, septic shock, ulinastatin, mortality, survival, randomized

## Abstract

**Background**: Sepsis poses a major global challenge in critical care medicine. While guideline-recommended interventions fail to reverse the core pathophysiological process of sepsis: inflammation–immune imbalance. Ulinastatin mitigates inflammatory cytokine storms. However, its impact on 28-day and 30-day survival rate in sepsis remains controversial, and dose-dependent efficacy has not been systematically clarified. **Methods**: This systematic review and meta-analysis adhered to the PRISMA guidelines. Randomized controlled trials published from February 2016 to September 2025 were identified through PubMed, Embase, Cochrane Library, and ClinicalTrials.gov. Adult patients (≥18 years) with sepsis (Sepsis-3 criteria: SOFA ≥ 2 + suspected infection) were included. Primary outcomes were 28-day and 30-day survival rates; secondary outcomes included inflammatory markers and prognostic scores. Data were pooled using RevMan 5.4.1, with RR for binary outcomes and mean difference for continuous variables. Heterogeneity was quantified by the I^2^ statistic, and publication bias was assessed via funnel plots and Begg’s/Egger’s tests. **Results**: In six included RCTs (*n* = 535), ulinastatin significantly improved survival rates at both 28 days (RR = 1.14, 95% CI: 1.01–1.29) and 30 days (RR = 1.39, 95% CI: 1.20–1.60), with a statistically significant interaction between time points (*p* = 0.04). Exploratory subgroup analyses suggested a “diminishing trend” with increasing daily doses, with 400,000 units appearing optimal. Treatment for >5 days was associated with better outcomes. The intervention also significantly lowered key inflammatory markers. Safety reporting was limited. Safety data were limited to one study (*n* = 96), reporting mild adverse events (rash, hypotension). **Conclusions**: A benefit has been shown with ulinastatin at 400,000 units daily, but not with higher doses. Treatment beyond 5 days may improve outcomes, though longer duration is not clearly better. Current evidence is limited by geographic bias, small samples, and insufficient safety data. More high-quality, multinational RCTs are needed to confirm the dose–response and long-term safety.

## 1. Introduction

Sepsis is an organ dysfunction syndrome resulting from dysregulated host response to infection, posing a major challenge in global critical care medicine. According to the Sepsis-3 criteria, there were approximately 48.9 million sepsis episodes worldwide, with an estimated 11 million sepsis-related deaths accounting for 19.7% of total global deaths [1]. This imposes a heavy disease burden and consumes substantial medical resources, making sepsis a priority concern in public health. As research advances, the core mechanism of sepsis has been gradually explored: a “inflammation-immune imbalance” triggered by pathogens, where early pro-inflammatory cytokine storms (e.g., TNF-α, IL-6) and subsequent exhaustion of anti-inflammatory responses jointly drive multi-organ dysfunction. Although guideline-recommended standard therapies (early antibiotics, fluid resuscitation, vasoactive drugs, etc.) can improve prognosis, they struggle to reverse this core pathological process. This underscores the need to prioritize inflammation–immune regulation to better enhance patient outcomes.

Studies have revealed that the inflammatory cytokine storm in sepsis primarily activates inflammatory gene expression via the MAPK signaling pathway, hypoxia-inducible factor 1α (HIF-1α), and nuclear factor erythroid 2-related factor 2/Kelch-like ECH-associated protein 1 (Nrf2/Keap1) pathway [2]. Ulinastatin, a serine protease inhibitor, inhibits the MAPK signaling pathway to cut off the amplification effect of inflammatory signals, thereby reducing the production of inflammatory factors while exerting anticoagulant and cytoprotective effects [3], Thus, ulinastatin is regarded as a highly promising adjuvant therapy for sepsis.

In clinical practice, although some studies indicate that ulinastatin can improve inflammatory markers, its effects on 28-day and 30-day survival rates in sepsis remain highly heterogeneous. For instance, some earlier studies found no significant improvement in 28-day and 30-day survival rates with ulinastatin [4,5], whereas recent studies suggest it may be a promising therapeutic agent for sepsis [6]. This discrepancy may stem from high study heterogeneity, such as differences in study populations and varying dosages of ulinastatin. Previous meta-analyses included studies conducted over an extended period; however, since the Surviving Sepsis Campaign (SSC) proposed Sepsis-3 in 2016 [7], new diagnostic criteria for sepsis have been established. Merging studies based on the two sets of criteria may not be appropriate. Secondly, previous studies evaluating the efficacy of ulinastatin in sepsis have largely neglected its dose–response relationship, leaving the optimal use of ulinastatin for maximizing benefits undefined. Therefore, conducting a further analysis is imperative.

Therefore, this study retrieved randomized controlled trials (RCTs) from 2016 to the present to explore the effects of ulinastatin on 28-day and 30-day survival rates and inflammatory factors in sepsis patients.

## 2. Materials and Methods

This meta-analysis was conducted in accordance with the Preferred Reporting Items for Systematic Reviews and Meta-Analyses (PRISMA) guidelines. The PubMed, Embase, ClinicalTrials.gov, and Cochrane Library databases were searched from 2016 to 15 February 2025, to identify RCTs. There were no language restrictions. This study has been registered in the International prospective register of systematic reviews (PROSPERO) with registration number 2025 CRD420251145330.

### 2.1. Literature Search

The Cochrane Library, PubMed, Embase, and ClinicalTrials.gov databases were searched. Additionally, the reference lists of the included articles were manually screened. The following keywords were used in the search: “sepsis,” “septic shock,” “ulinastatin,” “placebo,” “mortality,” “survival,” “28-day,” “ 30-day,” “ short-term,” and “randomized controlled trial” (Supplementary Materials Table S1).

### 2.2. Inclusion and Exclusion Criteria

The inclusion criteria were as follows: (1) RCTs or clinical trials; (2) intervention with Ulinastatin; (3) Adult (≥18 years) sepsis by Sepsis-3 (SOFA ≥ 2 + suspected infection); (4) Report 28-day or 30-day survival rate. Effect sizes were calculated using existing data. The exclusion criteria were as follows: animal studies, unpublished data, review articles, and conference abstracts.

### 2.3. Data Extraction

Two lead authors independently screened the titles and abstracts of the retrieved studies and reviewed the full texts to determine whether the studies met the inclusion criteria. Any discrepancies were resolved by the corresponding author. The following data were extracted into electronic spreadsheets: (1) first author’s name, (2) publication year, (3) country where the study was conducted, (4) study design, (5) number of participants in the intervention and control groups, (6) intervention protocol, (7) drug dosage, (8) duration of intervention, (9) age and gender of study participants, as well as 28-day or 30-day survival rate, inflammatory markers (including Interleukin-6 (IL-6), Procalcitonin (PCT), C-reactive Protein (CRP), Tumor Necrosis Factor-alpha (TNF-α), and Lactic Acid (Lac)), prognostic scores (such as Acute Physiology and Chronic Health Evaluation II (APACHE II) and Sequential Organ Failure Assessment (SOFA)), and adverse events, etc.

### 2.4. Quality Assessment

This meta-analysis employed Cochrane standards to evaluate the quality of included studies, with a focus on determining the levels of selection, performance, detection, attrition, and reporting biases across individual trials. Each domain was independently assessed to quantify the degree of bias within its specific scope. Study quality was appraised using the original Cochrane Risk of Bias Tool, specifically designed for RCTs [8].

Six key dimensions were evaluated: random sequence generation (selection bias), allocation concealment (selection bias), blinding of outcome assessment (detection bias), incomplete outcome data (attrition bias), selective reporting (reporting bias), and other potential biases. Each parameter was classified into one of three categories: low risk of bias (+), high risk of bias (−), or unclear risk of bias (±). For studies lacking explicit descriptions of randomization methods, the risk of bias is now classified as “unclear”; for allocation concealment and blinding, they were classified as “unclear” if the relevant information was not explicitly mentioned in the text; for selective reporting, registered trials were verified via platform checks and categorized as “high risk” if selective reporting was identified or “low risk” otherwise, while unregistered studies were classified as “unclear”; for data completeness, studies with an attrition rate below 15% were classified as “low risk”; otherwise, they were classified as “high risk”.

### 2.5. Statistical Analysis

Statistical analysis was conducted using Review Manager (version 5.4.1; Cochrane Collaboration, London, UK). The heterogeneity of studies was quantitatively assessed using the Higgins’s I^2^ index (with thresholds: 0–25% low, 25–50% moderate, 50–75% high, and >75% very high), and both fixed-effect and random-effects models were employed to summarize the effect size of controlled comparisons (to compare results under different assumptions of between-study variance). Using Stata 18.0 (StataCorp LLC, College Station, TX, USA) to draw a funnel plot, while simultaneously applying Begg’s test and Egger’s test for the analysis of publication bias.

For the primary binary outcomes—28-day and 30-day survival rates—the risk ratio (RR) with 95% confidence intervals (CIs) was selected as the effect measure, preferred for RCTs due to its intuitive interpretation of treatment effect (i.e., directly reflecting the ratio of survival probability in the ulinastatin group versus the control group). RR > 1 favors ulinastatin, indicating an improvement in survival. For the primary outcome analysis, we plan to explore dose–response relationships and other subgroup effects, and to ensure sufficient statistical power for these exploratory models, a decision was made a priori to potentially pool data from both time points. A test for subgroup interaction was planned to inform this decision. It was prospectively defined that if significant heterogeneity was suggested (interaction *p* < 0.05), pooling would proceed as an explicit methodological compromise strictly for exploratory purposes. Any findings from such pooled analyses are therefore considered hypothesis-generating, their limitations regarding potential introduced heterogeneity are acknowledged, and this is thoroughly discussed in the study limitations. For continuous variables, the mean difference (MD) with 95% CI was used. The effect size was calculated based on baseline comparability. If baseline data were comparable (defined as no statistically significant intergroup difference, *p* > 0.05, via independent t-tests), post-intervention means were compared using the MD. If baseline data were not comparable, change values were compared, and the MD of changes was pooled.

We planned subgroup analyses to explore sources of heterogeneity and conducted sensitivity analyses (e.g., leave-one-out method) to assess result robustness. All tests were two-tailed, and *p* < 0.05 was considered statistically significant.

## 3. Results

**Figure 1:**
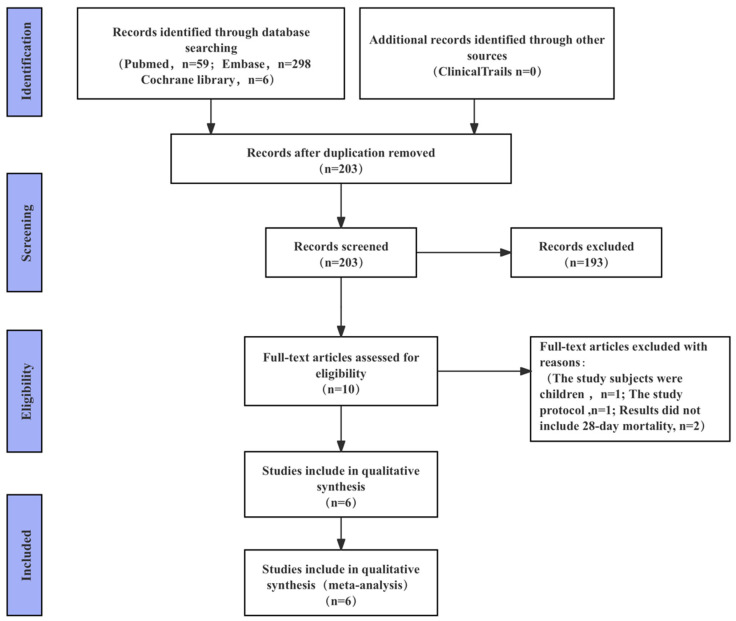
Flow diagram.

**Figure 2:**
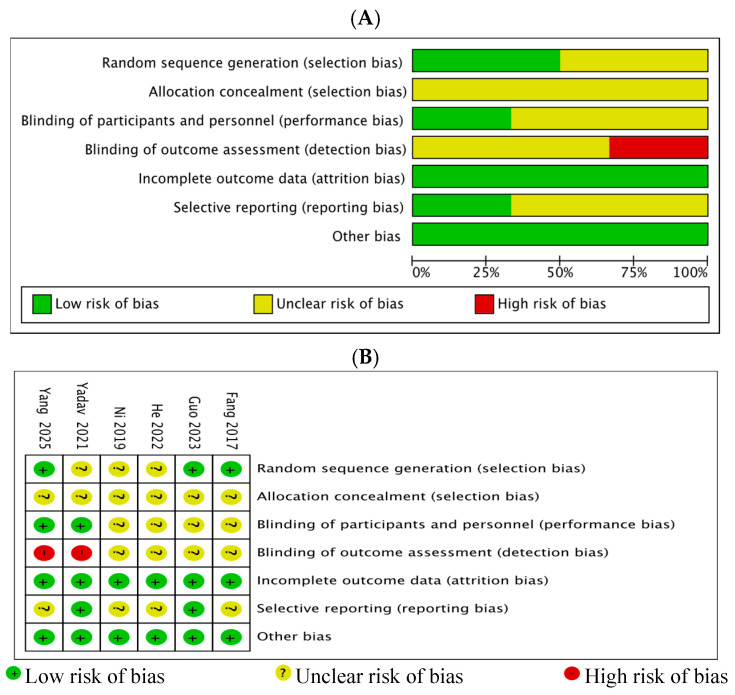
Assessment of risk bias. (**A**) summarizes the risk of bias across six studies using the original Cochrane Risk of Bias Tool. Each bar represents the proportion of studies rated as low (green), unclear (yellow), or high (red) risk for each bias domain listed on the *y*-axis. (**B**) displays the specific risk of bias assessment for each individual study: a green plus (+) indicates low risk, a yellow question mark (?) indicates unclear risk, and a red minus (−) indicates high risk [9,10,11,12,13,14].

This study ultimately included 6 trials comprising 535 participants; the majority of studies are from China [9,10,11,12,13]. The detailed literature search process is illustrated in Figure 1. The quality assessment results of the included studies are shown in Figure 2. Based on the risk-of-bias assessment, for random sequence generation, three studies clearly described the method (e.g., random number table) and were assessed as low risk, while the remaining three studies only mentioned “randomization” without specifying the procedure and were assessed as unclear risk; for allocation concealment, none of the articles mentioned the method, so all studies were assessed as unclear risk. Regarding blinding, two articles implemented blinding of participants (assessed as low risk) and the remaining four as uncertain risk, while two articles did not implement blinding of outcome assessors (assessed as high risk) and the remaining four as uncertain risk. For completeness of outcome data, all studies had a loss-to-follow-up rate below 15% and were assessed as low risk. For selective reporting, two articles registered in a clinical trial registry and reported all key outcomes (assessed as low risk), with the remaining four assessed as unclear risk. For other biases, all studies did not report potential confounding factors (e.g., baseline imbalances, conflicts of interest), so all studies were assessed as low risk. The clinical trials employed varying doses of ulinastatin over treatment durations of 3–7 days, with cumulative doses ranging from 1.8 to 6.3 million units. The trials participants were patients with sepsis or septic shock. The characteristics of the included clinical trials are presented in Table 1.

### 3.1. Primary Outcome

#### 3.1.1. 28-Day Survival Rate

**Figure 3:**
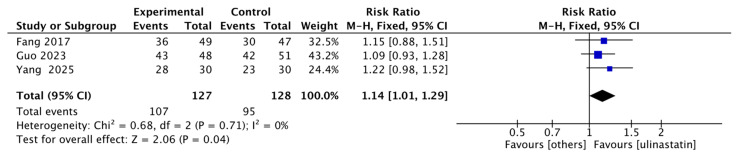
Forest map of 28-day Survival Rate. Each blue-black square in the figure represents the point estimate (risk ratio) of an individual study, with its horizontal width indicating the 95% CI for that estimate. The overall effect is represented by the black diamond at the bottom, where the center of the diamond corresponds to the pooled risk ratio (1.14), and its horizontal span shows the 95% CI for the pooled estimate [10,12,13].

Three studies reported either 28-day survival rate [10,12,13], with 127 patients in the ulinastatin group and 128 in the control group. The results showed that the 28-day survival rate in the ulinastatin group was 1.14 times higher than that in the other treatment groups, with 95% CI of [1.01, 1.29] and low heterogeneity (I^2^ = 0%). There was a significant difference (z = 2.06, *p* = 0.04) (Figure 3).

#### 3.1.2. 30-Day Survival Rate

**Figure 4:**
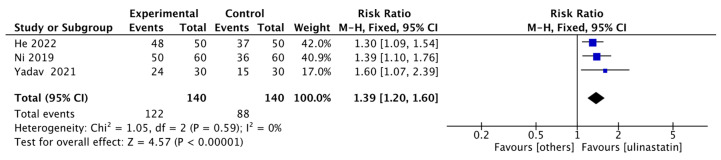
Forest map of 30-day Survival Rate. Each blue-black square in the figure represents the point estimate (risk ratio) of an individual study, with its horizontal width indicating the 95% CI for that estimate. The overall effect is represented by the black diamond at the bottom, where the center of the diamond corresponds to the pooled risk ratio (1.39), and its horizontal span shows the 95% CI for the pooled estimate [9,11,14].

Three studies reported either 30-day survival rate [9,11,14], with 140 patients in the ulinastatin group and 140 in the control group. The results showed that the 30-day survival rate in the ulinastatin group was 1.39 times higher than that in the other treatment groups, with 95% CI of [1.20, 1.60] and low heterogeneity (I^2^ = 0%). There was a significant difference (z = 4.57, *p* < 0.00001) (Figure 4).

#### 3.1.3. Interaction Analysis Between 28-Day and 30-Day Survival Rates

**Figure 5:**
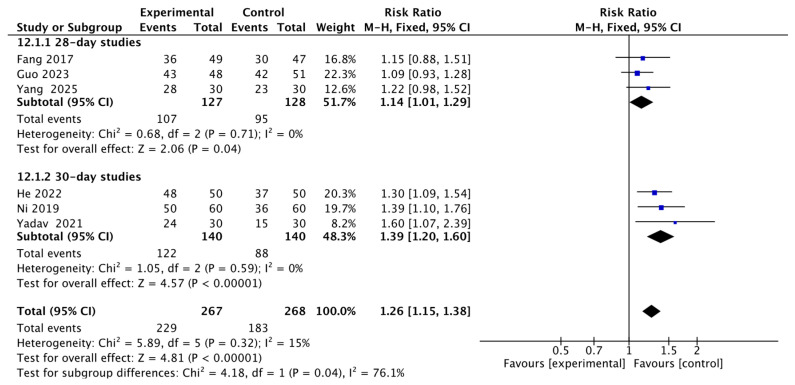
Forest plot of the subgroup analysis for interaction between 28-day and 30-day survival rates. Each blue-black square in the figure represents the point estimate (risk ratio) of an individual study, with its horizontal width indicating the 95% CI for that estimate. The overall effect is represented by the black diamond at the bottom, where the center of the diamond corresponds to the pooled risk ratio (1.26), and its horizontal span shows the 95% CI for the pooled estimate [9,10,11,12,13,14].

To statistically assess the appropriateness of pooling 28-day and 30-day survival data, and to investigate whether the time point serves as an effect modifier, we pre-specified an interaction analysis between these two endpoints. The test for subgroup differences yielded a statistically significant result (Chi^2^ = 4.18, df = 1, *p* = 0.04), with considerable heterogeneity (I^2^ = 76.1%) between the two subgroups. This significant interaction indicates that the effect of ulinastatin may differ meaningfully between the 28-day and 30-day time points. Consequently, while the pooled estimate across all studies provides an overall signal of benefit, the results for these two time points are presented separately in the following sections to reflect this heterogeneity (Figure 5).

#### 3.1.4. Exploratory Analysis of Dose–Response and Duration-Response Relationships

**Figure 6:**
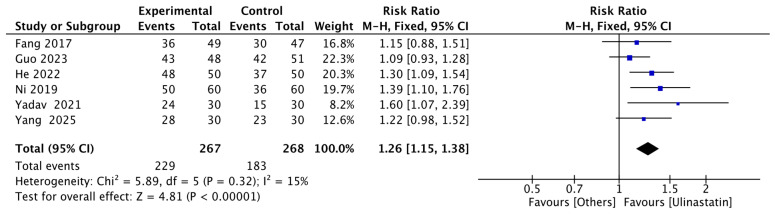
Forest map of 28-day or 30-day Survival Rate. Each blue-black square in the figure represents the point estimate (risk ratio) of an individual study, with its horizontal width indicating the 95% CI for that estimate. The overall effect is represented by the black diamond at the bottom, where the center of the diamond corresponds to the pooled risk ratio (1.26), and its horizontal span shows the 95% CI for the pooled estimate [9,10,11,12,13,14].

Given that each independent subgroup (28-day and 30-day survival subgroups) included only 3 studies, the limited sample size resulted in insufficient statistical power for reliable dose–response analysis within individual subgroups. In light of this constraint, we adopted the following compromise strategy: Although significant heterogeneity existed between the 28-day and 30-day subgroups (interaction *p* = 0.04), to explore the critical clinical question of ulinastatin’s potential dose–response and duration-response relationships, we proceeded with an exploratory analysis and hypothesis-generating approach by pooling 28-day and 30-day data into a composite endpoint of ‘28-day or 30-day survival rate.’ The results of dose and duration subgroup analyses based on this pooled data are presented herein. It must be emphasized that this analysis represents an initial signal exploration; its results may be biased by heterogeneity and thus require cautious interpretation. Validation through large-sample, homogeneous-design studies is warranted in the future. Six studies reported either 28-day or 30-day survival rate, with 267 patients in the ulinastatin group and 268 in the control group. The results showed that the 28-day or 30-day survival rate in the ulinastatin group was 1.26 times higher than that in the other treatment groups, with 95% CI of [1.15, 1.38] and low heterogeneity (I^2^ = 15.0%). There was a significant difference (z = 4.81, *p* < 0.00001) (Figure 6).

**Figure 7:**
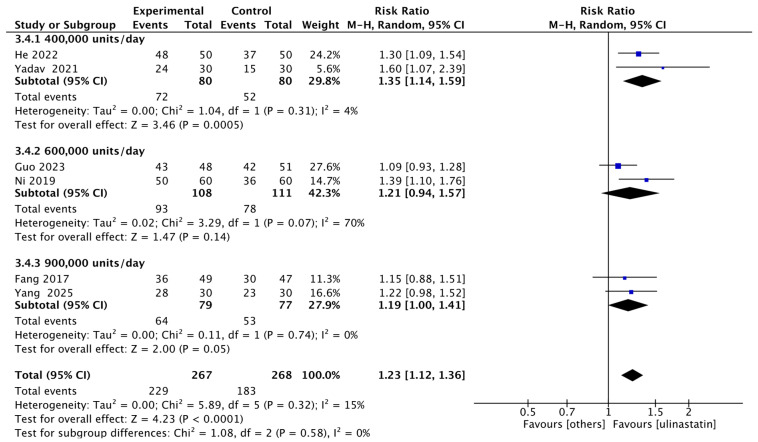
Exploratory subgroup analysis forest map of daily dose. This forest plot presents the pooled risk ratio of clinical events between different dosing groups of ulinastatin and the control group [9,10,11,12,13,14].

**Figure 8:**
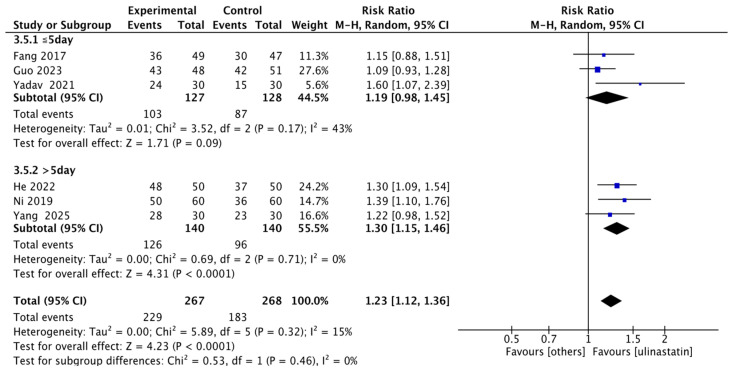
Exploratory Subgroup analysis forest map of duration. This forest plot presents the pooled risk ratio of clinical events between different duration groups of ulinastatin and the control group [9,10,11,12,13,14].

We then performed exploratory subgroup analyses based on this pooled data. For daily dose, we stratified into 400,000 units per day, 600,000 units per day, and 900,000 units per day. We found that: the 400,000 units/day group had a RR of 1.35 (95% CI: 1.14–1.59; I^2^ = 4%, *p* = 0.31), accounting for 29.8% of the total weight; the 600,000 units/day group had an RR of 1.21 (95% CI: 0.94–1.57; I^2^ = 70%, *p* = 0.07), accounting for 42.3% of the total weight; and the 900,000 units/day group had an RR of 1.19 (95% CI: 1.00–1.41; I^2^ = 0%, *p* = 0.74), accounting for 27.9% of the total weight (Figure 7). For duration of use, we stratified into >5 days and ≤5 days. The ≤5 days group had an RR of 1.19 (95% CI: 0.98–1.45; I^2^ = 43%, *p* = 0.17), accounting for 44.5% of the total weight; the >5 days group had an RR of 1.30 (95% CI: 1.15–1.46; I^2^ = 0%, *p* = 0.32) (Figure 8).

### 3.2. Secondary Outcome

#### 3.2.1. IL-6

**Figure 9:**
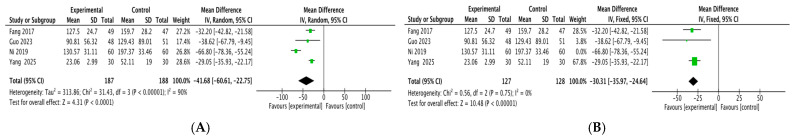
(**A**). Forest map of IL–6. The black diamond at the bottom denotes the pooled result from a random-effects model, showing a mean difference of −41.68 (95% CI: −60.61 to −22.75) and substantial heterogeneity (I^2^ = 90%). (**B**). Sensitivity analysis of IL–6. In the subsequent sensitivity analysis (after excluding the Ni 2019 study), the green squares for the remaining three studies are summarized by a black diamond from a fixed-effect model, yielding a mean difference of −30.31 (95% CI: −35.97 to −24.64) with negligible heterogeneity (I^2^ = 0%). In both plots, the size of each green square corresponds to the study’s weight in the analysis [10,11,12,13].

Four trials involving 375 participants calculated IL-6 [10,11,12,13]. Heterogeneity of these trials was substantial (*p* < 0.00001, I^2^ = 90%); therefore, a random-effects model was utilized. The result signified that there was statistically significant difference between ulinastatin group and other group (MD = −41.68, 95% CI: −60.61 to −22.75; *p* < 0.00001) (Figure 9A). Sensitivity analysis revealed that excluding any single study did not alter the results, indicating the robustness of the findings. After excluding the study by Ni, heterogeneity decreased to 0%. Using the fixed-effects model for analysis, the ulinastatin group was found to have a MD of −30.31 (95% CI: −35.97 to −24.64; *p* < 0.00001) (Figure 9B).

#### 3.2.2. PCT

**Figure 10:**
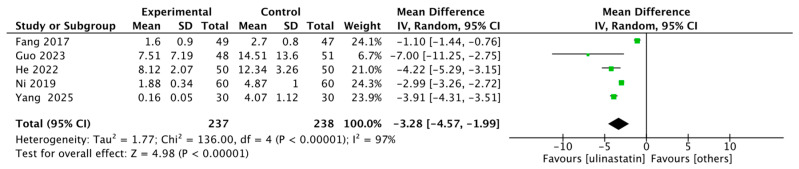
Forest map of PCT. Each green square denotes the point estimate and 95% CI for an individual study, weighted by its size, and the black diamond represents the overall effect MD = −3.28, 95% CI: −4.57 to −1.99), which favors ulinastatin despite considerable heterogeneity (I^2^ = 97%) [9,10,11,12,13].

Five studies reported either PCT [9,10,11,12,13], with 238 patients in the ulinastatin group and 268 in the control group. The ulinastatin group had an effect size of MD = −3.28, with 95% CI of [−4.57, −1.99] and high heterogeneity (I^2^ = 97.0%). There was a significant difference (z = 4.98, *p* < 0.00001) (Figure 10).

#### 3.2.3. CRP

**Figure 11:**
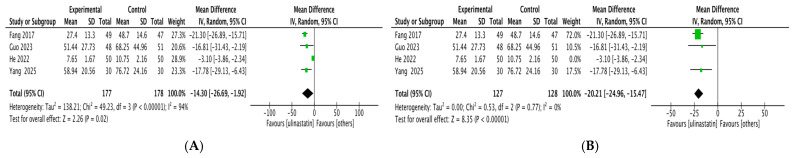
(**A**). Forest map of CRP. The black diamond at the bottom denotes the pooled result from a random-effects model, showing a MD of −14.30 (95% CI: −26.69 to −1.92) and substantial heterogeneity (I^2^ = 94%). (**B**). Sensitivity analysis of CRP. In the subsequent sensitivity analysis (after excluding the He 2022 study), the green squares for the remaining three studies are summarized by a black diamond from a fixed-effect model, yielding a MD of −20.21 (95% CI: −24.96 to −15.47) with negligible heterogeneity (I^2^ = 0%). In both plots, the size of each green square corresponds to the study’s weight in the analysis [9,10,12,13].

Four studies reported either CRP [9,10,12,13], with 177 patients in the ulinastatin group and 178 in the control group. The ulinastatin group had an effect size of MD = −14.30, with 95% CI of [−26.69, −1.92] and high heterogeneity (I^2^ = 94.0%). There was a significant difference (z = 2.26, *p* = 0.02) (Figure 11A). Sensitivity analysis revealed that after excluding the study by He, the heterogeneity I^2^ = 0%, and the effect size MD was −20.21, with 95% CI of [−26.69, −1.92]. There was a significant difference (z = 8.35, *p* < 0.00001) (Figure 11B).

#### 3.2.4. TNF-α

**Figure 12:**
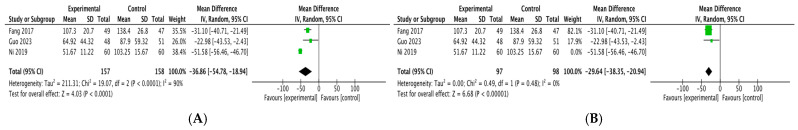
(**A**). Forest map of TNF–α. The black diamond at the bottom denotes the pooled result from a random-effects model, showing a MD of −36.86 (95% CI: −54.78 to −18.94) and substantial heterogeneity (I^2^ = 90%) (**B**). Sensitivity analysis of TNF–α. In the subsequent sensitivity analysis (after excluding the Ni 2019 study), the green squares for the remaining three studies are summarized by a black diamond from a fixed-effect model, yielding a MD of −29.64 (95% CI: −38.35 to −20.94) with negligible heterogeneity (I^2^ = 0%). In both plots, the size of each green square corresponds to the study’s weight in the analysis [10,11,12].

Three studies reported either TNF-α [10,11,12], with 157 patients in the ulinastatin group and 158 in the control group. The ulinastatin group had an effect size of MD = −36.86, with 95% CI of [−54.78, −18.94] and high heterogeneity (I^2^ = 90.0%). There was a significant difference (z = 4.03, *p* < 0.0001) (Figure 12A). Sensitivity analysis revealed that after excluding the study by Ni, the heterogeneity I^2^ = 0%, and the effect size MD was −29.64, with 95% CI of [−38.35, −20.94]. There was a significant difference (z = 6.68, *p* < 0.00001) (Figure 12B).

#### 3.2.5. Lac

**Figure 13:**
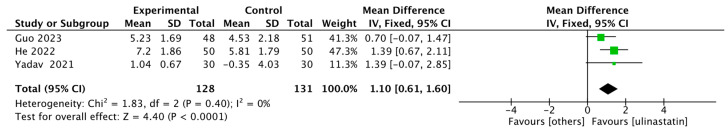
Forest map of Lac. Each green square in the figure represents the point estimate (MD) of an individual study, with its horizontal width indicating the 95% CI for that estimate. The overall effect is represented by the black diamond at the bottom, where the center of the diamond corresponds to the pooled MD = 1.10 (95% CI: 0.61 to 1.60) [9,10,14].

Three studies reported either Lac [9,10,14], with 128 patients in the ulinastatin group and 131 in the control group. Since the baseline Lac data were not comparable between the intervention and control groups, change values were adopted to calculate the effect size. The ulinastatin group had an effect size of MD = 1.10, with 95% CI of [0.61, 1.60] and no heterogeneity (I^2^ = 0%). There was a significant difference (z = 4.40, *p* < 0.0001) (Figure 13).

#### 3.2.6. APACHE II

**Figure 14:**
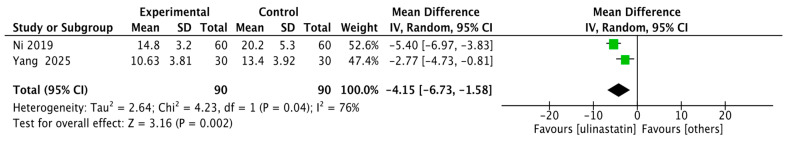
Forest map of APACHE II. Each green square in the figure represents the point estimate (MD) of an individual study, with its horizontal width indicating the 95% CI for that estimate. The overall effect is represented by the black diamond at the bottom, where the center of the diamond corresponds to the pooled MD = −4.15 (95% CI: −6.73 to −1.58) [11,13].

Two studies reported either APACHE II [11,13], with 90 patients in the ulinastatin group and 90 in the control group. The ulinastatin group had an effect size of MD = −4.15, with 95% CI of [−6.73, −1.58] and high heterogeneity (I^2^ = 76.0%). There was a significant difference (z = 3.16, *p* = 0.002) (Figure 14).

#### 3.2.7. SOFA

**Figure 15:**
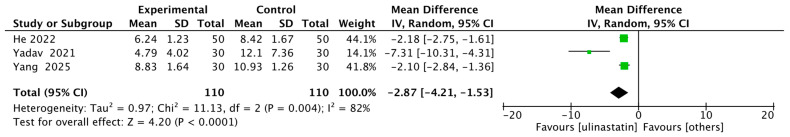
Forest map of SOFA. Each green square in the figure represents the point estimate (MD) of an individual study, with its horizontal width indicating the 95% CI for that estimate. The overall effect is represented by the black diamond at the bottom, where the center of the diamond corresponds to the pooled MD = −2.87 (95% CI: −4.21 to −1.53) [9,13,14].

Three studies reported either SOFA [9,13,14], with 110 patients in the ulinastatin group and 110 in the control group. The ulinastatin group had an effect size of MD = −2.87, with 95% CI of [−4.21, −1.53] and high heterogeneity (I^2^ = 82.0%). There was a significant difference (z = 4.20, *p* < 0.0001) (Figure 15).

### 3.3. Length of Stay in the ICU

**Figure 16:**
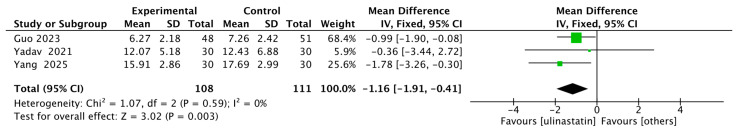
Forest map of length of stay in the ICU. Each green square in the figure represents the point estimate (MD) of an individual study, with its horizontal width indicating the 95% CI for that estimate. The overall effect is represented by the black diamond at the bottom, where the center of the diamond corresponds to the pooled MD = −1.16 (95% CI: −1.91 to −0.41) [10,13,14].

Three studies reported either length of stay in the ICU [10,13,14], with 108 patients in the ulinastatin group and 111 in the control group. The ulinastatin group had an effect size of MD = −1.16, with 95% CI of [−1.91, −0.41] and no heterogeneity (I^2^ = 0%). There was a significant difference (z = 3.02, *p* = 0.003) (Figure 16).

### 3.4. Safety of Ulinastatin

Only the study by Fang reported the safety of ulinastatin [12], involving 96 patients. In the ulinastatin group, 1 patient developed a rash, 1 had mild hypotension, and 1 experienced paroxysmal atrial fibrillation.

### 3.5. Sensitivity Analyses

We performed sensitivity analyses by sequentially excluding each individual study during the evaluation of both primary outcomes and drug safety. The results demonstrated that the exclusion of any single study did not significantly alter the pooled estimates.

### 3.6. Publication Bias

**Figure 17:**
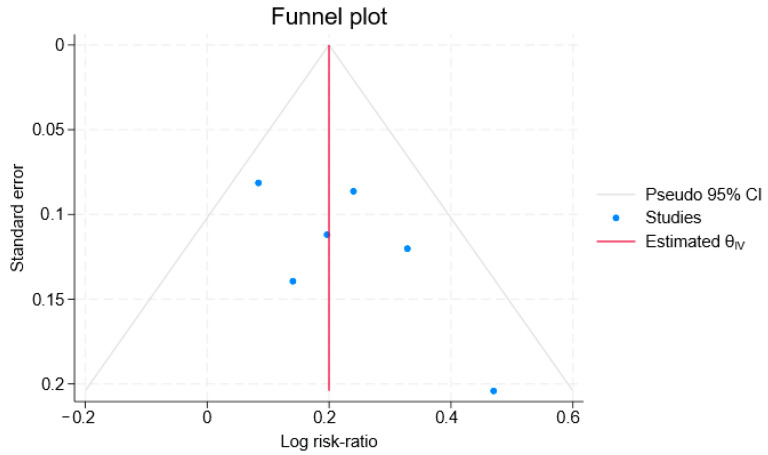
Funnel plot assessing publication bias for 28-day and 30-day survival rates. The blue dots represent individual included studies, plotted by their effect size (log risk ratio) on the *X*-axis against their precision (standard error) on the *Y*-axis. The solid red vertical line indicates the overall pooled effect estimate (θ_IV_).

We conducted an assessment of publication bias, and the result demonstrated a symmetrical funnel plot, indicating a low risk of bias in this study (Figure 17). Begg’s test (*p* = 0.26) and Egger’s test (*p* = 0.14) did not indicate any small-study effects for 28-day/30-day survival rate. It is worth noting that, as this meta-analysis included only six randomized controlled trials, the statistical power of using methods such as Begg’s test or Egger’s test to assess publication bias is limited. Nevertheless, a funnel plot was still generated for visual inspection, though its interpretation should be approached with caution.

## 4. Discussion

The management of sepsis remains a formidable challenge in critical care, necessitating novel therapeutic strategies that target the underlying dysregulated host response. While conventional therapies address the infectious insult, a significant gap exists for agents that effectively modulate the ensuing hyperinflammation and cytokine storm [15]. This meta-analysis fills this gap by providing, to our knowledge, the first comprehensive evaluation of the dose–response relationship of ulinastatin—a potent anti-inflammatory and immunomodulatory agent—in sepsis under the contemporary Sepsis-3 framework.

Our primary finding demonstrates that ulinastatin significantly improves the 28-day or 30-day survival rate in patients with sepsis. This result is consistent with the known anti-inflammatory properties of the drug and corroborates previous findings suggesting its potential benefit [16]. Delving deeper, two methodologically insightful observations emerged. First, the improvement in survival rate was more pronounced at 30 days compared to 28 days. This suggests that the anti-inflammatory and organ-protective effects of ulinastatin may require time to fully translate into a survival advantage, potentially by mitigating later-phase complications. This finding supports standardizing 30-day survival as the primary endpoint in future sepsis trials to better capture the full therapeutic benefit. Second, and crucially, our formal test confirmed a significant interaction between the 28-day and 30-day survival subgroups. This indicates that the timing of outcome assessment is not merely a logistical detail but a potential effect modifier, reflecting the dynamic nature of the host response and underscoring the paramount importance of endpoint standardization.

Subgroup analyses suggested that a daily dose of 400,000 units and a treatment duration exceeding five days were associated with optimal patient prognosis. An intriguing “diminishing trend” in the improvement of survival rate was observed with increasing daily doses, though the benefit remained statistically significant. It must be emphasized that these subgroup analyses are exploratory in nature. The findings on dose and duration should be interpreted as generating hypotheses for future research rather than providing conclusive evidence. These results may be influenced not only by clinical heterogeneity but also by the methodological heterogeneity introduced by pooling different endpoints. Consequently, all subgroup findings require cautious interpretation, and validation through large-scale, prospectively designed studies is warranted.

Meanwhile, the generalizability of our findings is constrained by the limited number of included studies and a significant geographic bias, as all trials were conducted in Asia. Furthermore, high-quality evidence regarding the safety profile of ulinastatin remains scarce. The limited sample size also precluded a powerful analysis of secondary outcomes like SOFA score reduction. Therefore, we consider the current evidence to be primarily exploratory and indicative of a promising survival benefit. Regarding safety, retrospective studies report that adverse reactions associated with ulinastatin are mostly mild (e.g., constipation, rash, nausea, vomiting, and diarrhea), with fewer reports of other serious adverse events [17]. Therefore, future research should prioritize large-scale clinical trials to investigate the initiation timing, optimal daily dose, and treatment duration of ulinastatin. Such efforts will provide more robust evidence for sepsis management and ultimately improve patient outcomes.

In light of our findings, future research should prioritize a definitive, large-scale, multinational randomized controlled trial. Such a trial should be prospectively designed to investigate the optimal dosing regimen (e.g., initiating at 400,000 units/day) and treatment duration (>5 days), using a standardized 30-day survival rate as the primary endpoint. Additionally, further investigation into the mechanisms by which ulinastatin modulates inflammatory markers such as IL-6 and TNF-α, potentially through pathways involving NF-κB signaling [18,19,20], will provide a deeper mechanistic rationale for its clinical use. Such concerted efforts will provide the robust evidence necessary to translate these promising signals into definitive clinical practice guidelines, ultimately improving survival outcomes for patients with sepsis.

### Limitations

This study has several limitations that should be considered when interpreting the findings. First, the generalizability of the results is constrained by a significant geographic bias, as all six included RCTs were conducted in Asian populations, predominantly in China. Consequently, the findings, which may reflect region-specific healthcare resources and clinical practices, require validation in more diverse ethnic and healthcare settings. Second, the relatively small total number of included trials (*n* = 6) and sample size (*n* = 535) limit the statistical power for robust subgroup analyses. Although our literature search was systematic, the limited scope of databases and the potential omission of unpublished or ongoing studies may introduce a risk of publication bias. While the funnel plot appeared symmetrical, the statistical power to reliably detect asymmetry was low with only six studies. Third, the observed high heterogeneity in the analyses of secondary outcomes, such as inflammatory markers and clinical scores, suggests underlying clinical or methodological variations not fully explained by our analysis; these results should therefore be considered exploratory. Finally, a key methodological limitation stems from the pooling of 28-day and 30-day survival data. Although this approach was adopted to enhance statistical power for a preliminary assessment, the significant interaction identified between these time points (*p* = 0.04) indicates that the choice of endpoint acts as an effect modifier. While the pooled estimate provides an overall signal of benefit, this methodological compromise introduces heterogeneity and underscores the need for future trials to standardize a primary survival endpoint.

## 5. Conclusions

A beneficial signal has been observed with ulinastatin at a daily dose of 400,000 units, but there is no strong evidence that higher doses provide additional benefit. A treatment duration exceeding 5 days may be beneficial for improving patient outcomes, but it cannot be inferred that longer use leads to greater benefit. However, existing evidence is limited by geographical bias (predominantly from China), small sample sizes, and inadequate safety data. High-quality RCTs from more countries and regions are urgently needed to validate the dose–response relationship and long-term safety.

## Figures and Tables

**Table 1:** Characteristics of the included studies.

First Author	Year	Country	Trial Type	Duration (Days)	Total Dose (U)	Intervention	Control	Participate	Primary Outcome	Journal
He [9]	2022	China	RCT	7	3.36 million	Ulinastatin+ Norepinephrine	Norepinephrine	septic shock	30-day survival rate	Pak J Pharm Sci
Guo [10]	2023	China	RCT	3	1.80 million	Ulinastatin	Placebo	septic shock	28-day survival rate	Immun Inflamm Dis
Yadav [14]	2021	India	RCT	5	2.00 million	Ulinastatin	HATS	sepsis septic shock	30-day survival rate	Journal of Clinical and Diagnostic Research
Ni [11]	2019	China	RCT	7	4.20 million	Ulinastatin+ Hemodialysis	Hemodialysis	sepsis septic shock	30-day survival rate	Acta Medica Mediterranea
Fang [12]	2017	China	RCT	5	4.50 million	Ulinastatin+ Hemodialysis	Hemodialysis	sepsis	28-day survival rate	Tropical Journal of Pharmaceutical Research
Yang [13]	2025	China	RCT	7	6.30 million	Ulinastatin	Placebo	sepsis	28-day survival rate	Drugs R D

Note: HATS means hydrocortisone, vitamin C and thiamine.

## Data Availability

The data supporting the findings of this study are available from the corresponding author upon reasonable request.

## Supplementary Materials

The following supporting information can be downloaded at: https://www.mdpi.com/article/10.3390/jcm15072682/s1, Table S1: PRISMA 2020 checklist. Reference [21] are cited in the supplementary materials.
